# Framing menstrual regulation as a pathway to advancing reproductive autonomy in restrictive legal contexts

**DOI:** 10.3389/frph.2026.1793216

**Published:** 2026-05-04

**Authors:** Jakaria Hossain, Grace Sheehy, Brittany Moore, Allison Campbell

**Affiliations:** Ipas (United States), Chapel Hill, United States

**Keywords:** abortion access, Bangladesh, global health policy, menstrual regulation, reproductive rights, reproductive autonomy

## Abstract

While 60 countries have expanded legal abortion access over the past three decades, implementation gaps persist, necessitating innovative approaches beyond conventional law reform. This paper examines the concept of menstrual regulation, which refers to actions taken to bring back a late period for an individual who may or may not be pregnant. Although only formally institutionalized in Bangladesh, the practice of menstrual regulation has resonance to how women in diverse contexts perceive delayed menses and possible early pregnancy. In this paper, we trace the evolution of the menstrual regulation program in Bangladesh, from an emergency post-war intervention to its integration in the national family planning program, through periods of service expansion and decline, and explore the substantial impacts and persistent challenges faced in its implementation. We then examine the global evidence beyond Bangladesh, which reveals related practices taking place across Asia, Africa, Latin America, and Europe, demonstrating widespread cultural resonance and policy applicability. We argue that by working within the ambiguity of early pregnancy status and using linguistic ambiguity and strategic policy framing to navigate legal restrictions, menstrual regulation offers a pragmatic, contextually responsive pathway to reproductive autonomy when conventional abortion law reform is untenable. However, advocates must ensure that menstrual regulation serves as a step toward, not a substitute for, comprehensive abortion law reform and full realization of reproductive rights.

## Introduction

Unsafe abortion is a preventable yet leading cause of maternal morbidity and mortality, with 97% of unsafe abortions occurring in low- and middle-income countries (1). Beyond restrictive laws, social and cultural factors create a spectrum of barriers to abortion care that significantly impact health and well-being and violate international human rights. While 60 countries and territories have expanded legal indications for abortion in the past 30 years (2), gaps between legal access and implementation persist, revealing a need for innovative approaches.

Pregnancy is usually considered a straightforward binary (pregnant/not pregnant), with actions taken to prevent pregnancy considered contraception, and those taken after pregnancy has been confirmed considered abortion. However, this dichotomy overlooks nuances in how many women understand and navigate fertility and reproduction. The time between a woman's menstrual periods can often represent a grey area where she could or could not be pregnant, especially when it is too early to take a pregnancy test or a period is potentially delayed for reasons beyond pregnancy (such as stress, illness or malnutrition). For many women, this ambiguity shapes their conception of pregnancy and is meaningful in supporting their reproductive decision-making (3). Within this ambiguity inhabits the practice of menstrual regulation, which refers to actions taken to bring back a period for women with recent delayed menses, without confirmation of pregnancy via laboratory test or ultrasound (4).

Bangladesh is the only country to have formally institutionalized MR in its family planning program. The ambiguity of pregnancy status allows for the possibility of menstruation being delayed for reasons other than pregnancy and allows MR to remain legally permissible despite using the same methods as abortion care, which is highly restricted. This paper examines MR's evolution, impacts and challenges, and assesses its potential applicability in other contexts where abortion is legally restricted or highly stigmatized. Drawing on an extensive literature review and analysis, we offer insight into how a pragmatic health intervention like MR can bolster reproductive autonomy in the absence of conventional abortion law reform.

In this paper we engage with MR as both established clinical practice and as a strategic framing. There is a tension to advocating for expanding reproductive autonomy by promoting MR as a service that is distinct from abortion care, while acknowledging it involves the use of abortifacient technologies to address menstrual irregularity, and continuing to strive for the full realization of reproductive rights. Our central argument is that MR's core innovation — working within the ambiguity of early pregnancy status and using linguistic ambiguity to navigate legal restrictions — offers a pragmatic, contextually responsive pathway to reproductive autonomy when conventional abortion law reform is untenable. We advance this argument not as a substitute for law reform, but as a complement to it.

## Overview of menstrual regulation in Bangladesh

As the sole example of a country where MR has been formally introduced as a health service, it is useful to understand how this approach was introduced in Bangladesh, how it has been implemented, and the successes and challenges it has faced in this context.

### From emergency intervention to national policy (1971–1979)

During Bangladesh's liberation war, thousands of women experienced pregnancies as a result of rape (5). Following an appeal from the Bangladeshi government, and a temporary waiver of the Penal Code restriction on abortion, international non-governmental organizations (NGOs) sent a small team of doctors to the country to provide emergency abortion services for these women. In 1971, Pathfinder funded the National Postpartum Program to provide contraceptive services and abortion care using manual vacuum aspiration (MVA), and provide training for providers (5). Bangladeshi doctors, government officials, and international specialists developed an MR training program, and a 1975 study of 1,068 MR cases demonstrated its safety and acceptability (6).

As evidence and acceptance of MR grew, further attempts were made to liberalize the abortion law but religious and political opposition blocked reform. The government ultimately deemed it unnecessary to take further measures to legalize abortion due to MR's availability, and formally integrated MR into the national family planning program in 1979 (7, 8). The framing of MR as a family planning method was essential to its successful introduction and staying power, protecting it from resistance to abortion liberalization; however, as described below, this protection also brought challenges to its full realization.

### Service expansion amidst political constraints (1980s–2000s)

By the early 1980s, MR had become a key component of the national family planning program, and a central intervention to reduce abortion-related mortality and morbidity (9). While political shifts in the late 1980s and growing social conservatism under military government rule impeded explicit abortion policy dialogue, MR provision was able to continue quietly, largely without backlash, due to its framing as a family planning method; enabling continuity of care, at times at the expense of visibility.

This progress was accompanied by considerable implementation gaps, including commodity constraints, trained provider shortages, and poor facility conditions, particularly in rural areas (10). Following the 1994 International Conference on Population and Development (ICPD), donor priorities began shifting toward broader reproductive health initiatives, while international funding decreased and government ownership weakened, stalling MR growth through the 2000s (11). National data demonstrated this deterioration, with declining rates of MR and service-providing facilities between 2010 and 2014, particularly in rural areas (12). Further assessments suggested that institutional visibility and donor prioritization had waned, raising concerns about sustainability (13). The U.S. Global Gag Rule further constrained international financing, prompting NGOs to reduce MR engagement or redirect resources to maternal health programs; a pattern unfortunately likely to repeat in the coming years.

### Expansion of methods and provision of services (2010–present)

Local and international NGOs pushed the Bangladeshi government to address gaps in service delivery, leading to a series of efforts to improve access to MR in recent years. The 2014 National Menstrual Regulation Services Guidelines established universal standards for MR service delivery, aligned with WHO recommendations (8). The cadre of providers who could offer MR services was expanded over time to include nurses (as of 2013) and midwives (as of 2018), and the permitted limit was extended in 2015, with trained midlevel providers authorized to offer MR services up to ten weeks after the last menstrual period, and doctors authorized up to twelve weeks (14, 15). In 2013, the Directorate General of Drug Administration authorized domestic manufacturing of the mifepristone-misoprostol combination pack for MR, enabling a local supply of the medication and formally introducing menstrual regulation with medicines (MRM) into the national MR program in 2014, publishing MRM guidelines in 2015 (16). MR services were included in the Essential Service Package in 2016 and in 2024 a national strategy to scale up comprehensive MR services was launched to make them more widely accessible (17).

### Impact of Bangladesh's MR program

Bangladesh's MR program has delivered substantial health and economic gains. In 2014, an estimated 430,000 MR procedures were performed (18), representing a considerable number of potential unsafe abortions averted. In the Matlab region, maternal deaths attributable to unsafe abortion fell from 16% in 1986–1990 to 9% in 2001–2005, demonstrating MR's potential to reduce abortion-related mortality when services are made available (19, 20). The economic case for MR has also been compelling; a 2010 study found that the cost of providing MR was 40% of the cost of treating moderate abortion complications and 13% of the cost of treating severe complications in tertiary-level facilities, making provision of this service more cost-effective than providing post-abortion care for serious complications (21).

MR has also demonstrated its value as a pragmatic and flexible model for treating menstrual irregularity and possible pregnancy in low-resource emergency settings. When nearly 700,000 Rohingya refugees arrived in Cox's Bazaar in 2017, MR services were quickly established in refugee camps through coordinated government, NGO and civil society partnerships (22, 23). This demonstrates MR's value for emergency settings where needs for sexual and reproductive health care are high, while reproductive health services and abortion care are often limited due to legal restrictions, lack of funding, or perceptions that provision is not feasible (22).

### Challenges of the MR program in Bangladesh

Despite the advances made since MR's introduction, persistent challenges remain. The incidence of unsafe abortion and associated complications in Bangladesh remain high and have increased according to the most recently available data; an estimated 1.2 million induced abortions occurred in 2014, nearly triple the number of MR services provided, with an estimated 257,000 women treated for abortion-related complications in 2014 alone (18, 24). At the same time, MR services have declined; public health facilities permitted to offer MR services that were actually offering MR dropped from 57% to 42% between 2010 and 2014, while actual procedures declined 34% (24).

Several factors have contributed to these challenges. Service delivery challenges – some specific to MR and some consistent with health service delivery more broadly – have hampered expansion and availability of MR, including shortages of trained providers and commodities, substandard health facility conditions, and persistent rural-urban inequities. MR provision has been impeded by information asymmetry and persistent stigma. A 2011 study found that 30% of women of reproductive age were unaware of MR (25), and confusion about its legal status discouraged many from seeking this service (26). This confusion is compounded by provider misinformation and stigma. Knowledge of cutoffs according to timing of the last menstrual period can range widely; many providers were unclear about the specific limits for different cadres, leading to unnecessary referrals or denial of care (27). An estimated 27% of women seeking MR services were turned away, often for reasons unrelated to medical or legal eligibility, such as marital status, age, or lack of husband's consent (20, 24, 26).

Across Bangladesh, community attitudes toward MR vary widely, influenced by cultural, religious, socioeconomic and gendered factors, with implications for access. Some providers refuse to provide MR due to religious convictions or personal moral judgments (27, 28). Provider-driven stigma, coupled with confusion about MR's legal status, inhibits many women from seeking this service openly and as a result, many struggle to obtain trustworthy information and must navigate a patchwork of price points (29). This stigma contributes to information gaps about the cost of MR, which some providers take advantage of. Though MR is officially free of charge in the public sector, some providers illegally charge fees, and drug sellers often charge variably depending on the client's situation (52), while “brokers” work to direct women to informal clinics for a commission (27). Thus, despite being framed as a service distinct from abortion care and integrated within the family planning program, MR continues to face much of the stigma and service delivery challenges associated with abortion care.

These challenges highlight a central tension of the MR approach: while it can facilitate the availability of care for people facing a delayed menstrual period, it may also obscure its purpose and legality in the process, creating confusion among providers and clients alike. Stigma surrounding MR further impedes access. Thus, any country attempting to implement MR policy as Bangladesh has must grapple with the challenge of balancing the framing that makes it politically feasible, with the limitations this framing places on how fully MR can deliver on its potential.

## Moving beyond Bangladesh: is MR a feasible policy alternative in other countries?

While Bangladesh is the only country where MR is official policy, research from numerous settings has established its resonance globally, demonstrating how MR-like approaches have emerged organically in environments where abortion is legally restricted, or access is constrained by stigma or bureaucratic barriers. Notably, the concept of MR has cultural and linguistic resonance with how women across diverse settings experience fertility, menstruation and pregnancy. Several clear patterns emerge from the cross-cultural evidence: cultural and linguistic resonance with MR-like concepts; provision of early medication without pregnancy confirmation; and the perception among women that early pregnancy management can be a process that is distinct from abortion.

### Cultural and linguistic resonance of MR outside Bangladesh

Research from Malaysia, Nepal, India, and Indonesia has demonstrated that practices to regulate or bring back a late period have salience beyond Bangladesh, and are often perceived as being distinct from abortion care. In Malaysia, research has documented women describing the use of herbs or medicines to “cleanse” or “discard” a pregnancy (30), while in Nepal, prior to the formal introduction of medication abortion, healthcare practitioners reported allopathic medicines sold in markets that were marketed for “menstrual regulation” (31). Population-based research in Rajasthan, India revealed that 3.3 out of every 1,000 women reported having taken action in the past year to bring back their period when they were worried they may be pregnant, predominantly using home-based remedies (32). In Indonesia, some women preferred to refer to pregnancy termination as menstrual regulation, while some providers considered MR to be distinct from abortion care and displayed more supportive attitudes toward MR than abortion (33, 34).

The evidence from West Africa also makes a compelling case for the relevance of MR beyond Bangladesh; studies in Nigeria, Côte d’Ivoire, Ghana, and Senegal demonstrate both the prevalence and social acceptance of MR as a concept (32, 35, 36). Population-based survey research in Cote d’Ivoire and Nigeria documented one-year MR rates of 20.6 and 22.6 per 1,000 women, respectively (32); higher rates than those most recently reported in Bangladesh (24), and similar to MR rates reported in Ghana (37). In Cote d’Ivoire and Nigeria, most women in a nationally representative survey perceived MR and pregnancy removal to be distinct concepts, and generally considered MR to include more ambiguous situations in which a pregnancy was not confirmed (35). Qualitative research in Senegal similarly reported that women and youth largely regarded MR as a route to reinstate a missed period and noted that it could soften legal or ethical qualms that often accompany abortion (36). Research in Nigeria in the 1990s and early 2000s also documented women's use of various medicines and procedures to “clean” the womb and promote a regular menstrual cycle (38). The scale and organic nature of MR-like practices in West Africa suggests that formal policy could build on an existing and resonant cultural framework.

In Latin America, qualitative research in Mexico, Colombia, Ecuador and Peru similarly found that some women perceived use of medication abortion as akin to period regulation rather than abortion (39). This was particularly relevant if they were early in pregnancy, which contributed to their perception that they were not having an abortion. In Morelos, Mexico, this practice, known as “bringing down the period” was embedded within broader beliefs about menstrual health, where a missing period was understood as an indicator of illness that required treatment; remedies ranging from herbal treatments to hormonal injections were administered, and if menstruation resumed, it was assumed that the woman had not been pregnant (40). Research on abortion practices from several Caribbean islands found various turns of phrase used by providers and patients instead of the word abortion (41). Providers described their use of the term “menstrual regulation” rather than abortion and often blurred the lines between providing an abortion and treating a miscarriage in their diagnosis and medical charting. This evidence suggests that the linguistic dimensions of MR have resonance far beyond Bangladesh.

### Provision of early abortion without clinical confirmation in the US and Europe

In the United States and Europe, MR-like approaches have emerged as a pragmatic response to restricted abortion access. Although these contexts differ markedly from the highly restricted settings discussed above, they demonstrate that even where abortion is legal, services focused on menstrual regularity or early provision without pregnancy confirmation can support access, and they offer evidence on safety and acceptability that is relevant to making the broader case for MR.

In the United States a growing body of work has found considerable interest in and support for “missed period pills”, where medication abortion is taken when a pregnancy is suspected but not clinically confirmed (42, 43). A recent paper argued that MR could provide an important route around restrictions such as state-mandated waiting periods, reducing wait times and allowing people to seek care earlier than they might qualify for an abortion (44). Meanwhile, research on very early medical abortion in Europe indicates that the administration of medication abortion before ultrasound confirmation corresponds with women's preferences for expedited, more discreet, and less stigmatized services (45). During the COVID-19 pandemic, the UK government permitted the provision of medication abortion via telemedicine without a pregnancy test for up to 10 weeks of pregnancy (46). Recent studies found this option was safe, effective, and well-received by patients (47, 48). These examples demonstrate that MR-like service models are not only culturally resonant across diverse settings, but safe and feasible.

## Global policy and advocacy implications

The evidence reviewed above suggests that MR's core innovation — working within the ambiguity of early pregnancy and deploying linguistic flexibility to navigate legal and social constraints — holds transferable value across highly diverse settings. It resonates in contexts of formal legal restriction, in contexts with significant access barriers, and in settings where women themselves draw meaningful distinctions between regulating their menstrual cycle and having an abortion. This section considers the conditions necessary for MR to be a viable policy approach, how it relates to the broader reproductive rights agenda, and what the current global policy environment means for its prospects.

MR may be most feasible to introduce in settings where it has existing cultural and linguistic resonance; the evidence from West Africa, South and Southeast Asia, and Latin America suggests that the concept of bringing back a late period often has existing resonance that MR policy can build on. Absent this familiarity, building resonance locally will likely require in-depth research and strong community engagement before policy adoption is feasible. The existing legal environment surrounding abortion is also pertinent – MR may be a more feasible route than legal reform in settings with legal restrictions, but its introduction may be more feasible in contexts with existing legal indications for abortion. Finally, Bangladesh's experience has highlighted the challenges of implementing any health service when health system capacity is constrained; successful implementation of MR will require trained providers, steady supply of commodities, and sufficiently prepared facilities.

The expansion of reproductive health services to include MR aligns with international human rights frameworks that recognize reproductive autonomy as fundamental to human dignity and wellbeing, including the ICPD Program of Action's recognition of reproductive autonomy and CEDAW's mandate to eliminate discrimination against women. Integrating MR into family planning services could help facilitate governments’ duty to ensure healthcare access for all, and expand reproductive rights amidst persistent legal, cultural and social constraints (49–51). Framing MR as a family planning service in Bangladesh has allowed it to survive political pressure, but it has also contributed to its invisibility as a reproductive health service. If MR is considered as a policy option in new contexts, decision-makers should weigh whether integration into family planning programs offers the best strategic protection, or whether a broader sexual and reproductive health framing that can more explicitly acknowledge MR's role in fertility regulation better serves long-term goals.

Finally, the current global reproductive health environment is fraught due in part to a well-resourced anti-rights movement, major funding cuts, and the current US administration's rollback of global health assistance, with clear exclusions for reproductive health as well as an expanded Global Gag Rule. Amidst this context, and the historical chilling effect such measures have had on provision of abortion and other reproductive health services, MR could face similar consequences as in the past; however, it could also serve as an even more strategic entry point within existing legal and political constraints.

Importantly, while MR can support abortion access in the absence of legal reform, there is a risk that it may reduce political momentum for abortion law liberalization, as illustrated in Bangladesh, or further perpetuate abortion stigma by being positioned as a more morally acceptable alternative to abortion care. Thus, advocates must balance the immediate practical advantages of expanding MR access with ongoing legal reform and stigma reduction efforts, ensuring that MR is a step toward, not a substitute for, the full realization of reproductive rights.

## Conclusion

Menstrual regulation has the potential to be a pathway toward advancing reproductive autonomy, and resonates with women across diverse contexts. By working within the ambiguity of early pregnancy status and using linguistic ambiguity and strategic policy framing to navigate legal restrictions, menstrual regulation offers a pragmatic, contextually responsive pathway to reproductive autonomy when conventional abortion law reform is untenable. However, advocates must ensure that MR serves as a step toward, not substitute for, comprehensive abortion law reform and the full realization of reproductive rights.

## Data Availability

The original contributions presented in the study are included in the article/Supplementary Material, further inquiries can be directed to the corresponding author/s.
